# Effect of Alternative Preservation Steps and Storage on Vitamin C Stability in Fruit and Vegetable Products: Critical Review and Kinetic Modelling Approaches

**DOI:** 10.3390/foods10112630

**Published:** 2021-10-29

**Authors:** Maria C. Giannakourou, Petros S. Taoukis

**Affiliations:** 1 Department of Food Science and Technology, University of West Attica, 12243 Athens, Greece; 2 Laboratory of Food Chemistry and Technology, School of Chemical Engineering, National Technical University of Athens, 15780 Athens, Greece; taoukis@chemeng.ntua.gr

**Keywords:** conventional processing, post-processing conditions, Vitamin C degradation, hurdle technology, kinetic analysis, frequentist kinetics, stochastic methodology

## Abstract

Vitamin C, a water-soluble compound, is a natural antioxidant in many plant-based products, possessing important nutritional benefits for human health. During fruit and vegetable processing, this bioactive compound is prone to various modes of degradation, with temperature and oxygen being recognised as the main factors responsible for this nutritional loss. Consequently, Vitamin C is frequently used as an index of the overall quality deterioration of such products during processing and post-processing storage and handling. Traditional preservation methods, such as thermal processing, drying and freezing, are often linked to a substantial Vitamin C loss. As an alternative, novel techniques or a combination of various preservation steps (“hurdles”) have been extensively investigated in the recent literature aiming at maximising Vitamin C retention throughout the whole product lifecycle, from farm to fork. In such an integrated approach, it is important to separately study the effect of each preservation step and mathematically describe the impact of the prevailing factors on Vitamin C stability, so as to be able to optimise the processing/storage phase. In this context, alternative mathematical approaches have been applied, including more sophisticated ones that incorporate parameter uncertainties, with the ultimate goal of providing more realistic predictions.

## 1. Introduction

Acknowledging the importance of vitamins for human health, the chemical mechanisms; the kinetics of their degradation and the main factors affecting their loss during postharvest handling, industrial processing, distribution and storage have been thoroughly investigated, and there is a large database for a variety of perishable food products [1]. Among the water-soluble vitamins, Vitamin C has been demonstrated to contribute as an enzyme cofactor for biochemical reactions in the human body and to the necessary collagen formation to prevent scurvy development, to maintain the normal function of the immune system, to protect from oxidative stress and a number of other beneficial functions [2]. From a chemical viewpoint, L-ascorbic acid (AA) is a carbohydrate-like, highly polar and water-soluble compound whose acidic and reducing properties are attributed to the 2,3-enediol moiety. Among the L-ascorbic isomers, only L-dehydroascorbic acid (DHAA) has Vitamin C activity, whereas other isomers like L-isoascorbic acid, the C-5 optical isomer, D-ascorbic acid and the C-4 optical isomer have no nutritional value. In any case, AA and L-isoascorbic acid are widely used in the food industry for their reducing and antioxidative activity as food ingredients (e.g., for preventing fruit and vegetable enzymatic browning or for the curing of meats).

Considering the fact that Vitamin C is not produced in the human body, there is a daily referenced intake on an approximate level of 80 mg/day that should be provided through an appropriate diet, with this value being significantly affected by gender, age, health status and individual consumer lifestyle (e.g., smokers vs. non-smokers) [3]. As a result of being a basic nutritional element, sometimes also deliberately added into certain foods during their industrial manufacturing, its loss during different types of processing and subsequent storage has gained considerable interest in the relative literature [4,5,6,7,8,9,10]. Looking at the mechanism of Vitamin C degradation, it is generally accepted that ascorbic acid (AA) reacts through two major paths, the most common one being in the presence of oxygen (“aerobic pathway”), which leads to the formation of dehydroascorbic acid (DHAA), which can then follow different modes of degradation [11,12,13]. In the absence of oxygen (“anaerobic pathway”), L-ascorbic acid degrades without being oxidised first, and thus, DHAA is not formed. Based on this mechanism, besides a temperature effect, the roles of oxygen, oxidative agents and catalyst presence in L-ascorbic acid degradation mechanisms and kinetics have also been extensively studied [8,13,14].

In the current literature, there is abundant evidence (as will be described in this article) that Vitamin C is a sensitive, water-soluble compound considered to be prone to severe deterioration, especially during the conventional processing techniques (e.g., thermal treatment and drying), with extreme conditions leading to a rapid loss. In this context, novel techniques or an appropriate combination of preservation steps have been investigated, so as to alleviate the negative effects of the most popular industrial methods. On the other hand, despite what has been generally considered and accepted a priori, there are studies that show that processing may not be the main cause of Vitamin C loss; even if one achieves substantial control or selects the optimised conditions during the processing stages, the post-processing and handling conditions could be the determinant factor at the time of use. In this field, there remains a need for an in-depth review of more systematic studies trying to separately investigate and mathematically describe Vitamin C loss during each processing step, as well as during the subsequent distribution and storage of the perishable food products. This review paper aims at putting all these issues into the perspective of processes that are designed so as to mitigate the acknowledged sensitivity of Vitamin C.

When reviewing the current literature, it is evident that Vitamin C degradation is strongly affected by the food tissue in question, as well as the specific processing/storage conditions applied [15]. Therefore, it is deemed necessary to establish appropriate kinetic equations, so as to mathematically describe the relationship between the nutritional quality and the prevailing factors, such as temperature, time, oxygen concentration and moisture content. In this context, another topic addressed in this article is published kinetic models of AA degradation in foods and the main principles of kinetic analysis followed. From a meta-analysis on this data, there are different kinetic approaches, deriving various values for a number of kinetic parameters that can hardly be evaluated and compared. Alternative calculation algorithms are presented, stressing out the need to expand the models used in order to obtain more realistic predictions on the nutritional quality of a product based on the degradation of its Vitamin C content. In those more sophisticated approaches (frequently referred to as “stochastic models”), the uncertainty and the uncontrollability of the conditions and parameters are approached and incorporated into the integrated mathematical algorithm by using more advanced mathematical and statistical tools. To be able to implement and critically evaluate the pros and cons of each procedure, a representative case study is analysed based on the available published data [15,16,17].

## 2. Overview of Fundamental Information on Vitamin C

Under the chemical perspective, the term “Vitamin C” is globally used to describe any compound that is proven to possess a full or partial biological activity of L-ascorbic acid (L-AA). It includes esters of L-ascorbic acid, such as ascorbyl palmitate (100% relative activity), synthetic forms such as 6-deoxy-l-AA (33% relative activity) and the primary oxidised form of L-ascorbic acid, namely dehydroascorbic acid (L-DHAA) (80% relative activity). L-ascorbic acid is a lactone with an enediol group on carbons 2 and 3 (cyclic ester of a hydroxyl carboxylic acid) [18].

Vitamin C is a water-soluble micronutrient that has several important functions in human health. Since it possesses antioxidant properties, and it acts as a cofactor in various enzymatic and hormonal procedures, it is a major compound for the growth and repair of all tissues. It participates in the biosynthesis of catecholamines, L-carnitine, cholesterol, amino acids, collagen and some peptide hormones [19]. Vitamin C serves as a reducing agent that efficiently scavenges potentially damaging free radicals, protecting cells against oxidative damage caused by ROS. Therefore, it minimises oxidative stress and succeeds in controlling the inflammation and tissue damage closely related to immune responses. Based on all those properties, it has been extensively studied and acknowledged as an effective means of preventing atherosclerosis, certain types of cancer and cardiovascular diseases [20], and its effects on the nervous system have recently been explored [21,22].

The majority of plant and animal species have the ability to endogenously synthesise large quantities of Vitamin C from glucose and galactose through the uronic acid pathway; however, humans and other primates are incapable of doing so because of an enzyme deficiency (gulonolactone oxidase) as a result of a mutation [23]. The lack of Vitamin C causes scurvy, a pathological condition that may cause connective tissue damage, extreme fatigue, rheumatic pains in the legs, muscular atrophy and skin lesions and, in some cases, even death. As a result, natural sources of Vitamin C, such as fruits and vegetables, are necessary to supply human organism with this valuable compound. The intake recommendations for Vitamin C are provided in the Dietary Reference Intakes (DRIs) developed by the Food and Nutrition Board (FNB) at the Institute of Medicine (IOM) of the National Academies (formerly the National Academy of Sciences) [24]. DRI is the general term for a set of reference values used for planning and assessing the nutrient intakes of healthy people. Although depending on the age, gender and other special characteristics, the current recommended dietary allowance (RDA) for Vitamin C for adult men and women is roughly set at 75 mg/day and 90 mg/day, respectively, in order to maintain the maximum neutrophil concentration combined with a minimum urinary loss of ascorbate [24]. One should stress that, for special groups such as pregnant women, smokers or infants/adolescents, this reference value may substantially change.

As far as the side effects of excessive doses of supplemental Vitamin C, they are mostly related to gastrointestinal disturbances and to the effects of metabolites in the urinary system. For example, doses of 2 to 3 g/day may cause unpleasant diarrhoea. Additionally, oxalate, an end product of ascorbate catabolism, has been found to play an important role in kidney stone formation. Vitamin C may precipitate haemolysis in some people, including those with a known deficiency in glucose-6-phosphate dehydrogenase, paroxysmal nocturnal haemoglobinuria or other conditions where an increased risk of red cell haemolysis may occur.

**Figure 1:**
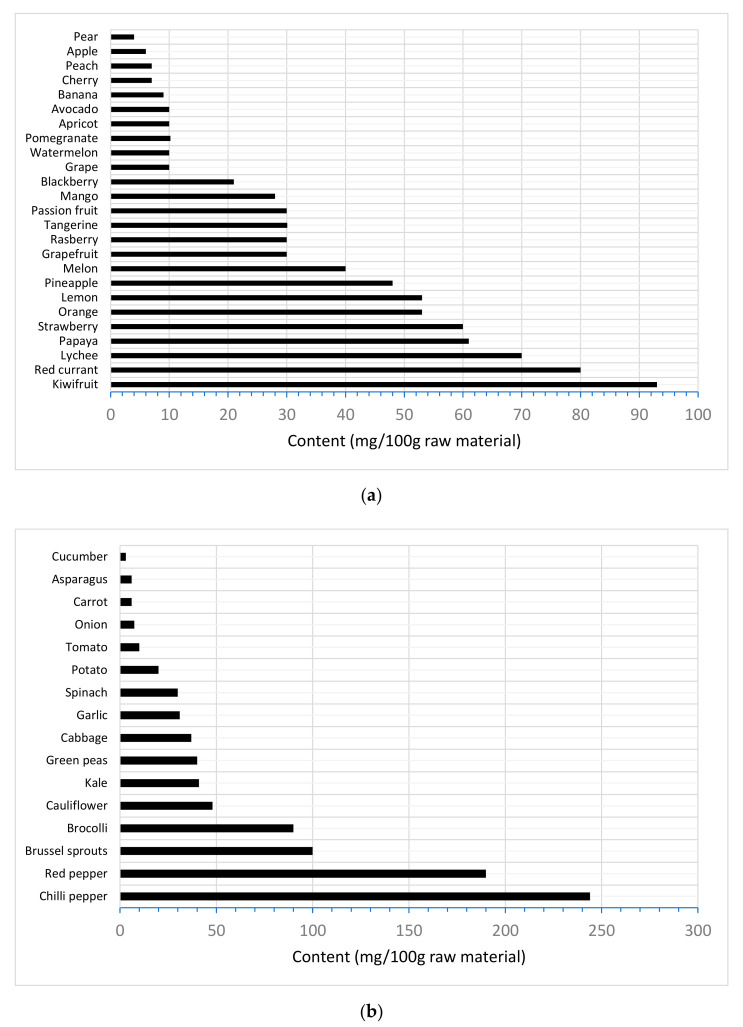
Mean content of Vitamin C in (**a**) fruits and (**b**) vegetables [2,18,27].

Fresh fruits and vegetables are considered as the most abundant sources of Vitamin C [25], and in Figure 1, an indicative value of the Vitamin C content of some of the most important species of plant origin is depicted. Citrus fruits and species such as cantaloupe, watermelon, berries, pineapple, strawberries, cherries, kiwi fruits, mangoes and tomatoes are particularly rich sources of Vitamin C. On the other hand, vegetables such as cabbage, broccoli, Brussels sprouts, bean sprouts, cauliflower, mustard, greens, peppers, peas and potatoes are equivalently considered as a good source of Vitamin C due to their high content but, especially, thanks to their availability for longer periods throughout the year. If Vitamin C is not naturally present in raw materials, it can be deliberately introduced into food as an effective antioxidant to prevent the enzymatic browning of fruit and vegetables, as it causes the reduction of the o-quinones, formed by the action of the polyphenol oxidase enzymes (PPO), back into their phenolic substrates [26]. Regarding legislative issues, ascorbic acid, as a food additive, is considered by the U.S. Food and Drug Administration as a generally recognised as safe antioxidant, mainly used to prevent browning actions.

## 3. Modes of L-Ascorbic Acid Degradation

**Figure 2:**
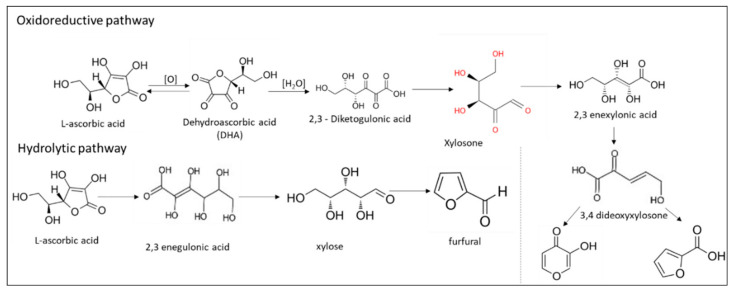
Alternative degradation mechanisms of L-ascorbic acid [28,29].

In the scientific literature, there are numerous studies about the different modes of Vitamin C degradation in complex food matrices, as well as on the factors that mostly affect such deteriorations. The main path of ascorbic acid degradation in liquid systems with a high water activity (>0.980) in the presence of oxygen is based on its oxidation to dehydroascorbic acid, which then degrades to 2,3-diketogulonic acid [28,29,30] (Figure 2).

Upon the hydrolysis of dehydroascorbic acid, the vitamin property is immediately lost. A water activity (a_w_) or moisture content increase has been found to accelerate the degradation of ascorbic acid [31], whereas the presence of Fe^3+^ ions accelerate both steps (oxidation/hydrolysis) of the Vitamin C degradation path [32]. On the other hand, cysteine facilitates the reconversion of dehydroascorbic acid to ascorbic acid. Ascorbic acid can also be degraded by an anaerobic pathway, where 2,3-diketogulonic acid is formed by hydrolysis of the keto tautomeric forms of L-ascorbic acid, and via a complicated path, various reductones, furfural and furancarboxylic acid are the final products recognised. In the latter path, ascorbic acid disintegrates without being first oxidised, which means that the well-known intermediate product of DHAA is not formed. Although rarely addressed, these two mechanisms of disintegration can be simultaneously observed, albeit at different rates [4].

As far as the factors that mostly affect Vitamin C stability, they include pH, light and temperature, as well as the concentration of trace metal ions, oxygen and degradative enzymes [33]. As it will be extensively discussed in a later section, high temperatures during thermal processing can significantly affect the rates of AA loss through an aerobic pathway. In the pH range between 2 and 4, the maximum stability is recorded [18], whereas both forms of AA and DHAA are known to be susceptible to degradation by both natural and UV light. Among the metal ions usually found in foods, cupric and ferric ions are known to be the strongest catalysts of L-AA oxidation. Another factor that negatively affects Vitamin C degradation in fruits and vegetables, besides the availability of oxygen species, is the accumulation of high quantities of CO_2_, such as, for example, in the case of the improper packaging of rocket leaves [34] or in the case of fresh-cut potatoes stored under MAP [35]. According to the authors, elevated CO_2_ levels within MAP packages of raw materials could accelerate the Vitamin C loss by oxidation of AA catalysed by ascorbate peroxidase. In Reference [36], orange juice packed in films of different oxygen permeability was kinetically studied in order to determine the extent of ascorbic acid loss due to oxygen as a function of time and temperature. The results confirmed that the rapid removal of oxygen was an important factor in retaining a higher concentration of ascorbic acid over long storage times, both at room and chill temperatures.

One of the principal factors affecting Vitamin C retention in food matrices is the natural occurrence of degradative enzymes, such as AA oxidase, cytochrome oxidase and peroxidase, which are readily inactivated through appropriate thermal treatment (often called “blanching”), during industrial applications.

On the other hand, several factors are found to protect Vitamin C from being oxidised, such as the presence of certain groups of phenols (e.g., flavonol) or sugars, which decrease the oxygen solubility and its availability, thus improving the Vitamin C retention [32].

## 4. Effect of Conventional and Novel Preservation Processes on Vitamin C

### 4.1. Effect of Thermal Processing on Vitamin C Retention

When addressing the major problem of extending the short shelf life of perishable fruit and vegetable products, traditional heat treatments, such as pasteurisation (at temperatures that usually do not exceed 100 °C) and sterilisation (at higher temperatures), are the reference preservation processes, although proven to significantly affect plant tissue nutritional properties. In Mieszczakowska-Frąc et al. 2021 [2], a brief outline of the impact of high temperatures was provided, where it was underlined that the use of intense conditions affects negatively the Vitamin C contents of fruits and vegetables. In the literature, there are numerous studies reporting on the impact of different thermal processing of fruit and vegetable products on the Vitamin C content, mainly pasteurisation [37,38,39,40,41] and sterilisation [42,43,44].

Looking critically at the results of this research, it is evidenced that Vitamin C retention is significantly affected both by the conditions of the processing (temperature and duration of the treatment) and the food matrix in question. However, qualitative generalisations, such as that thermal treatment is detrimental for the nutritional quality of fruits and vegetables, are of limited value, and quantitative analyses, translated into appropriate mathematical modelling, are often lacking.

As far as different methods of cooking are concerned, the most popular conventional methods (boiling, steaming and frying) and nonconventional ones (microwaving) were addressed in Soares et al. 2017 [45] for broccoli in terms of its detrimental effect on Vitamin C. Despite acknowledging that the extent of Vitamin C loss depends also on the process conditions and product properties, it was found (after an extensive review on the particular subject) that the greatest Vitamin C loss is measured after the combination of an initial boiling step in water (since Vitamin C is water-soluble) and then frying (38%) and after a single water boiling procedure (33%). Fried and microwaved broccoli demonstrate losses in the range of 24 and 16%, respectively. Steaming was found to be the technique with the best results regarding Vitamin C retention.

### 4.2. Effect of Low Temperatures on Vitamin C Retention

As pointed out in Reference [2], freezing has been considered as one of the most effective traditional preservation techniques for Vitamin C retention in fruit and vegetable products. Hot water or steam blanching, a short heat treatment, is frequently applied before freezing at the industrial level for some vegetables, which is found to slightly decrease water-soluble compounds, including L-ascorbic acid [46]. Alternatively, the use of chemicals (often called “chemical” blanching) can be effectively applied to avoid the heat degradation and water leakage of nutritional compounds [47,48]. In Xanthakis et al. 2018 [49], microwave-assisted blanching was proposed for frozen mangoes and shown to lead to a higher retention of the total Vitamin C in both low-temperature long-time and high-temperature short-time treatments. Nonetheless, obtaining the inactivation of the enzymes in all blanching procedures, this pre-treatment leads to an improved retention of Vitamin C during the subsequent frozen storage [50]. In order to improve the Vitamin C retention during frozen storage, a variety of pre-treatments, including an osmotic step, have been proposed, as will be explicitly discussed in the next section.

### 4.3. Effect of Water Reduction on Vitamin C Retention

Dehydration of the plant based raw materials is a popular industrial practice, aiming at obtaining easy-to-handle final products with extended preservation at ambient storage and lower transportation costs [51]. There are numerous studies that report the impact of different drying techniques on the nutritional degradation of fruits and vegetables, showing that the more severe the drying conditions, the greater the loss observed. Among the traditional drying methods, the solar drying of tomatoes was found to cause a 17% Vitamin C loss, with the subsequent storage at ambient conditions not leading to further degradation [52]. On the other hand, convective drying at high temperatures, frequently applied in the current industrial practice, causes a significant reduction of bioactive compounds, such as phenolic compounds and Vitamin C, degradation of sensory attributes, such as colour and texture and modification of structural attributes [53,54,55]. A good but higher energy and cost alternative to hot air drying can be freeze-drying, which is acknowledged as the optimum method from a quality retention perspective [56,57]. In the same context, milder drying procedures such as foam mat drying are found to be beneficial for bioactive compound retention [51], while vacuum microwave dryers and vacuum infrared dryers appear as promising drying techniques that have succeeded in decreasing the drying time and preserving the quality of the final dried foods [55]. Cold air drying, especially when it is combined with a mass transfer intensification step (such as ultrasound strengthening technology and/or a far-infrared radiation step) is proposed as a technique that mildly affects the nutritional quality of fruits and vegetables [58]. Nonetheless, in all the aforementioned studies, it was stressed that the matrix properties, in addition to the process conditions, determine the fate of Vitamin C. Additionally, hybrid drying procedures, including more than one intermediate moisture-reducing step, have been recently proposed in order to obtain a better retention of thermolabile compounds, an alternative that will be discussed in the next section.

### 4.4. Effect of Nonthermal Processes on Vitamin C Retention

Reviews of the effect of novel preservation technique processes (often classified as nonthermal to distinguish from conventional processes exclusively relying on heating-induced high temperatures) on the retention of Vitamin C have been provided in the literature [2,59,60,61]. Briefly, this sums up the following:

High-pressure processing, HHP (alternatively called high hydrostatic pressure or cold pasteurisation), is shown to increase the microbiological stability and reduce the enzyme activity, with an improved retention of bioactive compounds when compared to the traditional thermally processed products. There are numerous works that show the superior nutritional quality of HHP-processed products, focusing on Vitamin C, including fruit juices [62,63,64,65,66], vegetable smoothies [67] and a number of fruit and/or vegetable-based functional beverages [68]. A point to underline is that specific HHP treatments (pressure and temperature range, as well as processing time) are crucial for the effectiveness of the preservation method applied.

Ultrasound, being a mechanical wave that travels through the food medium, can cause physical destruction of the cells of the walls of the fruit, leading to an increase of the efficiency of the extraction of bioactive compounds and to an improved nutritional stability during storage. This treatment can also cause a reduction of the duration of the thermal process applied, a decline in the amount of dissolved oxygen, while it is considered to inactivate the native enzymes and possess an antibacterial effect. The benefits of this technology in the retention of Vitamin C were shown by Domínguez Avila et al. 2018 [68] for functional beverages, by Manzoor et al. 2020 [69] for sugarcane juice, by Ordóñez-Santos et al. 2017 [70] for gooseberry juice and by Aguilar et al. 2017 and Khandpur and Gogate 2015 [71,72] for different fruit juices. However, in most studies, sonication is used as a complimentary assisting step and not as a single preservation step, as it will be discussed later on in the context of the hurdle technology approach [58,73].

Pulsed electric field (PEF) processing is a nonthermal method that is based on the application of pulses of high voltage (usually more than 20 kV/cm) to liquid foods placed between two electrodes, causing the destruction of microorganisms and the modification of fruit and vegetable structures, assisting in the intensification of certain procedures [2]. The higher Vitamin C retention in PEF-processed products of plant origin, as compared to those subjected to traditional high-temperature treatments, is shown for fruit juices in References [74,75,76], for plant-based products in Reference [77] and for tomato juice [78]. This advantageous retention is found to still hold to a significant extent during the subsequent cold storage of PEF-treated products.

Cold plasma is a rather new (in food applications), green and promising technology, which is found to be effective in microbial inactivation, except for its acknowledged efficiency in the surface decontamination of food products and packaging materials. When applying cold plasma at atmospheric pressure on blueberries, the results on Vitamin C retention indicated its positive effect during the subsequent storage for 20 days at 25 °C [79]. According to the authors, the prolongation of the shelf life obtained may be attributed to the regulation of the ascorbateglutathione cycle and the increase of dehydroascorbate reductase, followed by an excited species of NO, generated by cold plasma. Based on these, the changes of the Vitamin C content may be due to the rate of regeneration of ascorbic acid. When the rate of regeneration of ascorbic acid by the ascorbate–glutathione cycle is higher than the rate of decay of ascorbic acid through its reaction with other plasma-generated reaction species, the Vitamin C content increases.

The impact of pulsed light (PL) treatment conditions on the Vitamin C content in Indian gooseberry (amla) juice was explored, and the results showed that a 61% better retention was obtained in comparison to the thermally pasteurised counterpart [80]. Similarly, the positive effect of the ozone treatment when compared to traditional pasteurisation was shown by Sroy et al. 2019 [81], who studied Vitamin C behaviour in cantaloupe melon juice immediately after treatment but, also, during refrigerated storage.

## 5. Effect of Combined Preservation Processes (Hurdle Technology) on Vitamin C

### 5.1. Preliminary Treatments Combined with Conventional Drying

Significant water removal via drying is one of the most popular industrial methods to extend the shelf life and the availability to consumers for seasonal and perishable products, such as fruits and vegetables. Given the negative effect of the conventional drying techniques on the nutritional quality of fruits and vegetables, a number of pre-treatments have been proposed to reduce the intensity of the main drying step and improve the stability of the final dried product. Some representative examples of such multi-stage procedures, deriving a dehydrated food item with a superior Vitamin C content, are presented in Table 1.

In Kowalska et al. 2020 [82], a comparative study on the effect of different drying techniques on the retention of Vitamin C in apples was presented, where osmotic dehydration was coupled either with freeze-drying or with a hybrid method, including conventional/microwave and vacuum drying. Besides proving a greater retention in freeze-dried samples, it was shown that the greatest losses of Vitamin C, occurring in the first period of drying, were mostly attributed to the high water content and not to the elevated temperatures applied. Amanor-Atiemoh et al. 2020 [83] compared the effectiveness of different pre-treatments based on the immersion in hypertonic solutions of glucose (OD) and the application of ultrasound (US) before pulsed-vacuum drying and showed that the reduction of the drying time obtained with the combination of ultrasound/osmotic dehydration processes was the main factor retaining the Vitamin C content. Based on the fact that this compound is heat-labile, it is expected that exposure to longer drying times may cause irreversible chemical changes that would lead to its gradual loss [84]. Another major cause of loss observed when prolonged processing times were used was reported by Roueita et al. 2020 [85] in dried kiwifruit pre-treated with osmotic dehydration and an ultrasound. This can be attributed to the water-soluble nature of Vitamin C and to the formation of microchannels during the cavitation process.

In Wiktor et al. 2019 [86], different pre-treatments were studied prior to cranberry osmotic and then conventional air drying. Amongst them, blanching, and especially HTST blanching, was found to be very effective in the inactivation of ascorbic acid oxidase (AAO), protecting the Vitamin C content. On the other hand, innovative techniques, such as the application of pulsed electric fields (PEF) or ultrasound, promote the leakage of the water-soluble vitamin into the osmotic solution due to the electroporation and sonoporation occurring. The negative effect of an ultrasound was also observed in dried apricots pre-processed with osmotic dehydration with or without sonication [87]. The loss of Vitamin C, in this case, is mainly attributed to its water solubility and leakage into osmotic solutions and thermal degradation during the final step of hot-air drying at 55 °C. In the same work, pectin-based coatings were also applied on apricot samples, where the highest retention of Vitamin C (46.5%) was obtained for samples pre-treated with OD and AA incorporated in the pectin-based coating. An extended Vitamin C loss was also measured in sweet potatoes pre-treated with an ultrasound, a negative effect attributed to the combined impact of the formation of free radicals by ultrasound waves and the leakage of water-soluble solids [88].

On the contrary, in the case of dried sweet potatoes, the authors showed a positive effect of the ultrasound effect, especially during short processing times, whereas the OD treatment with glucose was not equally effective, possibly due to extensive leakage or to the presence of dissolved oxygen on the osmotic solution [89].

In the majority of works, OD is applied as a pre-treatment, mainly for shortening the main drying step and lowering the water activity of the food tissue. Besides the advantage of osmotic dehydration in terms of lower energy consumption, reduced browning, improved texture and the colour and appearance of fruits, osmotic dehydration could also affect the nutritional value of the dried final products. The immersion of fruits in osmotic solutions for a relatively long time causes the leaching of water-soluble components such as minerals, vitamins and organic acids. To avoid losses of such compounds, these have been added to osmotic solutions to counteract the leaching effect of osmotic dehydration [90,91].

**Table 1:** Vitamin C degradation in dried fruits and vegetables using different modes of dehydration (hurdle technology).

Type of Dried Food	Type of Dehydration Method	Conditions of Process	Main Findings	Reference
Apple	Osmotic Dehydration/freeze drying	OD: sucrose and chokeberry juice concentrate (osmotic solution) at 60 °C for 120 min Freeze drying: frozen samples dried in a drying chamber at 63 Pa at a 25 °C hot plate temperature for 24 h.	High losses during OD in sucrose greater loss of Vitamin C was shown in the samples dried by the hybrid method than freeze-drying increase in Vitamin C when chokeberry juice concentrate is used from the diffusion of components between solution-fruit The greater the number of operations, especially thermal ones, the greater the degradation of Vitamin C.	[82]
Apple	Osmotic Dehydration/hybrid drying (convective-microwave/vacuum)	OD: sucrose and chokeberry juice concentrate (osmotic solution) at 60 °C for 120 min hybrid drying: convection for 3 h at 50 °C and an air velocity of 2 m/s. Then microwave (400 W and the pressure 3.5 kPa) vacuum drying. The process temperature was 70 °C, and the drying time was 6 min.		[82]
Apple	osmotic dehydration [OD], ultrasound [US], and ultrasound-assisted osmotic dehydration [UOD])/drying under pulsed vacuum dryer (PVD)	OD pretreatment: glucose solution (30%, *v*/*w*) in a ratio (1:4) at 30 ± 1 °C for 30 min Ultrasonic pretreatment: 20 kHz as frequency, 300 W/L as power density, 10 s (on) and 3 s (off) as pulsation time, 30 ± 1 °C as temperature, and 30 min UOD pretreatment: combination of the abovementioned conditions PVD: at constant temperature (70 °C), vacuum pressure duration (10 min, 10 kPa) and ambient pressure duration (5 min, 101 kPa) at pulsation ratio 10:5. The heating panel distance to the sample was 30 mm.	rate of Vitamin C decline was intensified in samples with longer processing time OD showed the fastest decline, followed by US at the end of the drying process, a 46.05, 31.28, and 25.95% retention was achieved for UOD, US, and OD, respectively	[83]
Kiwifruit	Ultrasound-assisted Osmotic Dehydration Pretreatment/Final Drying	Three osmotic solutions (sugar solution, grape syrup and mulberry syrup); 55 °Brix at ultrasonic bath at 27 KHz and 65 °C in three time periods of 20, 30 and 40 min. cabinet Final drying in a cabinet dryer machine at 50 °C to reach 20% moisture content	longer ultrasonic time decreased the amount of Vitamin C protective effect of sugar on Vitamin C	[85]
Cranberries	Cutting/Blanching/OD + sonication	Blanching (BL) at 90 °C for 5 min in distilled water. The ratio of water mass to the material was 2:1 Ultrasonic treatment: at the frequency of 21 kHz and total power of sonotrodes amounted to 180 W, at 23 °C, for 30 or 60 min in two osmotic solutions: 1. 61.5% of sucrose (S) 2. 30% of sucrose with 0.1% of steviol glycosides (S + G).	cutting (C) and blanching (BL) led to degradation of Vitamin C due to oxygen access blanching causes thermal disruption of cranberry skin and leakage of water-soluble solids (BL) caused a 22% decrease of Vitamin C Prolonged US times intensified Vitamin C loss	[92]
Cranberries	Traditional method: Cutting/Blanching/OD/conventional drying Innovatine method: Blanching + PEF/Ultrasound + OD/conventional drying	Blanching (BL) at 90 °C for 5 min in distilled water. The ratio of water mass to the material was 2:1 Ultrasonic treatment + OD: at the frequency of 21 kHz (US intensity equal 3.6 W/g of material) at 40 °C for 30 or 60 min in two osmotic solutions: 1. 61.5% of sucrose (S) 2. 30% of sucrose with 0.1% of steviol glycosides (S + G). Convective drying at 70 °C and air velocity of 2 m/s	Best retention with blanching US/PEF treatment caused the degradation of ascorbic acid or enhanced the activity of the enzyme of AAO	[86]
Apricot	OD (with and without sonication)/pectin based coatings /hot-air drying	OD: sorbitol of 35 °Brix at 55 °C for 30 and 45 min using a fruit/osmotic solution ratio of 1/4 (*w*/*w*) ultrasound treatment by a 1.71-kW probe ultrasound processor (25 and 35 kHz) for 30 and 45 min Coatings made of pectin, pectin + CA or pectin + AA solutions Hot air drying at 60 °C with air velocity of 1.5 m/s to reach a moisture content of approximately 19–20% (wet basis)	highest retention (46.5%) for samples pretreated with OD and AA incorporation in the coating increase in OD immersion time resulted in a pronounced decrease in Vitamin C content	[87]
Sweet Potatoes	OD or/and US/hot air drying	OD: 10 and 20% glucose for 10, 20, 30 or 45 min Ultrasound (US): frequency at 20 kHz, intensity of 0.2 W/cm, and temperature at 30 °C hot-air drying at 60 °C with a 25% relative humidity and 1.5-m/s air velocity until product moisture content <5% (wet basis)	highest Vitamin C content was observed in the combined treatment of ultrasound and glucose of US/GC-20% short pre-treatment times (10 and 20 min) did not cause any serious Vitamin C degradation	[89]
Mango	OD/microwave drying	Osmotic dehydration at 25◦C in 45◦BRIX sucrose solutions with and without 1% (*w*/*w*) calcium chloride (or 1% ascorbic acid Microwave drying dried at either 50 °C or 70 °C with air velocity of 0.2 m/s to a water activity of approximately 0.60	the addition of calcium or Vitamin C to the osmotic solution can improve Vitamin C retention	[90]
Broccoli	OD/microwave-assisted hot air drying	OD: sucrose solution of 54 °Brix at 30 °C Drying: power of the dryer set to 100 W, at 40, 50 and 60 °C and at the superficial air velocity of 1.4 m/s.	67% Loss of Vitamin C after 2-h immersion time during osmotic dehydration process Vitamin C was negatively correlated with drying temperatures under microwave-assisted hot air drying	[91]

### 5.2. Preliminary Treatments Combined with Osmotic Drying

Blanching is a high-temperature treatment usually applied to plant tissues, aiming mainly at inactivating their natural enzymes, which are responsible for the subsequent quality loss during further processing, storage and handling. However, especially when a water medium is used for blanching, the thermal disruption of plant skins occurs, and a significant leakage of water-soluble solids (including Vitamin C) may take place [92].

Ultrasound (US) is another process frequently applied prior to OD in order to accelerate mass transfer phenomena. In any case, in terms of nutritional quality, an US is found to enhance the Vitamin C loss, especially when prolonged sonication times are applied. This negative effect may be attributed to the formation of free radicals by ultrasound waves, which are known to initiate specific reactions that may influence the Vitamin C content within a plant tissue [93].

### 5.3. Preliminary Treatments Combined with Thermal Processing

Orange juice was submitted to electroreduction and electrooxidation treatments prior to pasteurisation, and Vitamin C retention was assessed at three storage temperatures, representing chill, ambient and abusive conditions [14]. The results showed the combined effect of (a) dissolved oxygen, which was substantially increased during water electrolysis, thus promoting oxidation reactions, and (b) temperature, with the elevated temperatures causing a more intense decrease of Vitamin C. An interesting finding was that, in the electrooxidised juice, Vitamin C degradation during storage was less pronounced, possibly due to its initial decrease during the electrolysis treatment, making ascorbic acid the main limiting factor for the aerobic degradation pathway in the subsequent storage. This is a well-documented example of how (and why) an initial nutritional decrease as a result of a particular pre-treatment may not only be counterbalanced but even proven as advantageous during the subsequent storage of a food, a case frequently occurring in the case of hurdle technology applications.

### 5.4. Preliminary Treatments Combined with Freezing

Strawberries were initially osmo-dehydrated, using different osmotic agents—namely, glucose, oligofructose and high DE maltodextrin [10]—before being frozen. A storage study was conducted on a range of sub-zero temperatures, and the results showed that dehydrofrozen samples exhibited a significantly improved Vitamin C retention, especially at temperatures below −12 °C. This improved stability could be attributed to the glass transition behaviour of the modified osmo-dehydrofrozen food matrix. Similar research was presented by Giannakourou and Taoukis 2003 [94], where green peas were submitted to osmotic dehydration in carbohydrate–salt syrups prior to freezing and frozen storage in a wide temperature range ending at –24 °C. The dehydrofrozen samples retained their L-ascorbic acid contents, with the rate of Vitamin C loss being reduced by as much as three-fold for peas osmotically pre-treated with oligofructose, trehalose and maltitol systems. The cryostabilisation observed was related to the glass transition temperature increase measured for the modified osmodehydrofrozen food matrix.

## 6. Modelling Vitamin C Degradation during Processing/Storage

Vitamin C is a relatively labile component, and therefore, many kinetic studies use it as an indicator of nutritional quality loss or an oxidative stress index during a process or as a result of post-processing handling [95].

Most studies measure the Vitamin C content in the raw material and, immediately after processing, through a single or a multi-process procedure (hurdle technology), as presented in the previous sections. There are a few examples in the literature where Vitamin C degradation was systematically investigated during processing or during the subsequent storage of the processed food under conditions that were appropriate for the specific type of food, i.e., frozen/chill storage or ambient conditions for dehydrated foods. From the literature analyses carried out so far, it has been demonstrated that each preservation procedure, either as a unique step or as a separate link in the whole processing chain, has a specific impact on Vitamin C loss that is strongly dependent on the food matrix properties and process/storage-specific conditions. Given this observation, it is deemed necessary to establish a relationship between the main factors of the processing/storage and Vitamin C retention for different types of final products (raw, dried, frozen and minimally processed) through appropriate mathematical models and modelling procedures.

This section summarises the published works on Vitamin C degradation kinetics, aiming at analysing the effects of the main parameters. Regarding the mathematical approach, a detailed presentation on the alternative kinetic models proposed for Vitamin C degradation was provided by Peleg et al. 2016 [5]. In this work, the primary models (i.e., equations describing changes of the Vitamin C content vs. processing/storage times) available in the literature include first- and zero-order equations, as well as Weibullian kinetics and combined models, that take into account both aerobic and anaerobic degradation paths; the secondary models mainly include Arrhenius, the simpler exponential model, the *D-z* approach (based on the thermobacteriological principles), the square root model, the Eyring–Polanyi and other mathematical formulations. The main purpose of this paper was to use an interactive software (http://demonstrations.wolfram.com/SimulatingAscorbicAcidDegradation/ (accessed on 27 August 2021)) with which these equations can be applied in simulations, aiming also at reducing the number of chemical determinations in storage studies. Apart from reporting the mathematical equations used and the main results obtained, at the end of this section, an overview of the alternative kinetic approaches will be briefly and schematically outlined in an effort to underline the importance of estimating not only the value of the kinetic parameters involved but also the degree of their variability (also expressed by their uncertainty, confidence intervals or errors). This methodology, based, in some cases, on a stochastic approach, can provide a more realistic prediction of the expected nutritional quality of a product under conditions that simulate the realistic distribution chain.

### 6.1. Effect of Processing Conditions on Vitamin C Loss

In Ghasemi et al. 2019 [96], Vitamin C degradation during tomato convective drying at 45, 60 and 75 °C was kinetically simulated by a numerical approach of heat and mass transfer processes. For nutritional deterioration, this study used models available in the literature where the Vitamin C content was a function of both the temperature and moisture content, with the latter playing a major role in vitamin loss, at different locations inside the tomato matrix (for example, at the central, inner and superficial nodes). The models had a good correlation with the experimental data, showing that, during dehydration, the highest concentrations of Vitamin C were obtained at the surface layer, which had the least moisture content, whereas the lowest value was obtained at the sample centre, with the highest moisture content. Yeasmin et al. 2021 studied the effect of mechanical drying on Vitamin C degradation in carambola tissue at isothermal conditions of 65, 60 and 55 °C, assuming a first-order reaction (Equation (1)) as the primary model, whereas the Arrhenius equation (Equation (2)) was used as a secondary model to show the temperature impact on the degradation rate constant. Based on the authors’ calculations, an *E_a_* of about 38.66 kJ/mole (9.24 kcal/g-mole) was estimated [47].

(1) $$C_{t}=C_{0}e^{-kt}$$

(2) $$\begin{array}{c} k=k_{0}\cdot e^{-\frac{E_{a}}{RT}}\;\mathrm{or}\\ k=k_{ref}exp\left(-\frac{E_{a}}{R}\left(\frac{1}{T}-\frac{1}{T_{ref}}\right)\right),\;\mathrm{when}\;a\;\mathrm{reference}\;\mathrm{temperature}\;\mathrm{was}\;\mathrm{used}. \end{array}$$

where *k* is the reaction rate (in (time units)^−1^, *C_o_* is the mean value of the initial Vitamin C concentration, *R* is the universal gas constant, *E_a_* the activation energy of the phenomenon (in kJ/mol), *T* is the absolute temperature of storage, *T_ref_* a reference temperature selected depending on the range of temperatures studied (in degrees K) and *k_ref_* is the reaction rate at that reference temperature.

Similarly, the kinetics of the nutritional quality changes during the winter jujube slice drying process were studied by Niu et al. 2021 at a temperature range between 55 and 70 °C [97]. In terms of the kinetic analysis, zero-order (Equation (3)), first-order (Equation (1)) and Weibull models (Equation (4)) were applied to fit the experimental data, with the latter being the most suitable one.


(3) $$C_{t}=C_{0}-kt$$

(4) $$C_{t}=C_{0}e^{-{\left(k_{a}t\right)}^{\beta }}$$

According to the Arrhenius equation, which was used to describe the temperature effect on the rate of Vitamin C loss, the activation energy, *E_a_*, was 63.78 kJ/mol.

Vitamin C loss during the pasteurisation of mango juice at isothermal conditions (at 60, 70, 80 and 90 °C) was kinetically described in Ogori et al. 2020, applying a zero-order equation, Equation (3) (as a primary model), and the Arrhenius model (as a secondary model) (Equation (2)) [98]. A rather low value of activation energy—namely, *E_a_* = 12.192 kJ/mol—was estimated. In a similar approach, the thermal degradation of L-ascorbic acid in rosehip nectar was investigated in Reference [99] at a range between 70 and 95 °C, who selected a first-order reaction to describe the Vitamin C loss vs. time, and the Arrhenius law as a secondary model, estimating an *E_a_* of approximately 55 kJ/mol. The authors also reported *Q*_10_, described by Equation (5), which is an alternative way of expressing the temperature dependence of the degradation rates, which was estimated near the absolute value of 2.


(5) $$Q_{10}={\left(\frac{k_{2}}{k_{1}}\right)}^{\frac{10}{T_{2}-T_{1}}}$$

The kinetics of Vitamin C loss in mangoes during osmotic dehydration (OD) were described by Sulistyawati et al. 2020 using a multi-response model, combining the rate of leaching and that of degradation, according to a set of equations, that accounts for the change of the Vitamin C content change within fruit tissue, as well as within the OD solution [100]. The results revealed that the degradation path during OD had the most important effect on Vitamin C loss compared to the one caused by leaching into the OD solution. However, the contribution of leaching becomes crucial when OD solutions are repeatedly used [101,102] in an effort to optimise the procedure and maximise the effectiveness of the osmotic medium.

Besides the processing temperature, the presence of oxygen has a major impact on Vitamin C degradation. Therefore, the impact of the partial pressure of oxygen on ascorbic acid degradation at canning temperatures was mathematically studied by Al Fata et al. 2018, assuming that ascorbic acid degradation is allowed to follow two pathways, the oxidoreductive one, which produces dehydroascorbic acid (DHAA), and the hydrolytic one by direct cleavage of the lactone ring of the ascorbic acid molecule [28]. Nth-order apparent kinetics were applied for Vitamin C degradation in a model system, with the value of the order changing depending on the partial pressure of oxygen (for example, *n* = 1 for anaerobic conditions), and the Arrhenius law was once more used, with an activation energy estimated (in the case of anaerobic conditions) at 67 kJ/mol. In some cases, the behaviours of ascorbic acid and DHAA were separately studied as functions of the temperature and oxygen concentrations, showing that the AA degradation increased with the temperature and oxygen concentrations, while DHAA behaved like an intermediate species, appearing and then disappearing. In that detailed kinetic analysis, a kinetic model was developed to describe the experimental data by two first-order consecutive reactions [103]. Those two reactions, for aerobic conditions, were also analysed in a detailed kinetic approach (using first- or other fixed-order kinetics as the primary models and the exponential secondary model) in Peleg 2017 (Equation (6)), who aimed at providing solutions in the case of realistic non-isothermal conditions, proposing the use of a user-friendly software [4].


(6) $$k=k_{ref}exp\left(c\left(T-T_{ref}\right)\right)$$

On the other hand, when oxygen was present in ascorbic acid degradation, the results showed that the apparent reaction rate constants deviated from the Arrhenius law, possibly due to the interference of more complicated reaction paths, or (alternatively) there could be an interface phenomenon between the heated media and the headspace gas.

The combined impact of the water content and temperature on the thermal degradation of ascorbic acid during drying was investigated by Frías and Oliveira 2001 in model systems using maltodextrin DE 12 as the base material [104]. The authors applied two alternative kinetic approaches, the one including the classical thermobacteriological equation (D-z approach), where the terms of D and z were considered to depend on the water content (Equations (7) to (9)), and the second applying WLF kinetics for the D-value (Equation (10)), assuming that the phenomenon is mostly governed by molecular mobility restrictions and the glass transition temperature range, *T_g_*. In the latter approach, the influence of the water content is incorporated into the integrated equation through the Gordon-Taylor equation (Equation (11)), giving rise to a more complicated model, which accounts for both temperature and water content changes during drying

(7) $$C=C_{0}10^{-t/D_{T}}$$

where *D_T_* (decimal reduction time) is defined as the time in minutes at a constant temperature required to reduce the initial concentration of a thermolabile substance by 90%:

(8) $$\log D_{T}=\log D_{T_{ref}}+\frac{T_{ref}-T}{z}$$

where *D_Tref_* is the decimal reduction time (in min) at the reference temperature, and z stands for the number of degrees °C required to change the *D*-value by one decimal log unit (°C). Giving an overall model based on the TDT model widely used in thermal processing (instead of the Arrhenius law), where the temperature is a function of time (non-isothermal case), Equation (9) is derived:

(9) $$\log\frac{C}{C_{o}}=-\int_{0}^{t}\frac{10^{\frac{T(t)-T_{ref}}{z(w)}}}{D(w)}dt$$

(10) $$\log\frac{D_{Tg}}{D}=\frac{C_{1}\cdot \left(T-T_{g}\right)}{C_{2}+\left(T-T_{g}\right)}$$

where *D_Tg_* is the decimal reduction time (in min) at the glass transition temperature, and *C*_1_ and *C*_2_ are the system-dependent coefficients. The glass transition temperature is calculated by the Gordon-Taylor equation (Equation (11)), assuming food as a bicomponent mixture:

(11) $$T_{g}=\frac{kwT_{g(water)}+(1-w)T_{g(maltodextrin)}}{kw+(1-w)}$$

Based on this “biomaterial science approach”, the overall model gives

(12) $$\log\frac{C}{C_{o}}=-\int_{0}^{t}\frac{10^{C_{1}C_{2}\left(T-T_{ref}\right)/\left(C_{2}-\left(T_{g}-T_{ref}\right)\right)\left(C_{2}+\left(T-T_{g}\right)\right)}}{D(w)}dt$$

An alternative, stochastic approach to predict the changes in the Vitamin C concentration during the processing of canned green beans via a probabilistic and modular process model is presented by Rigaux et al. 2016, accounting for the statistical uncertainty and/or variability of factors such as the initial Vitamin C concentration and environmental factors, parameters that were incorporated within the estimation algorithm [44].

### 6.2. Effect of Post-Processing Storage on Vitamin C Loss

Besides processing, the Vitamin C retention is significantly affected—even to a greater extent, in some cases—from the subsequent storage conditions of the processed food. In this section, a brief outline of the kinetic modelling methodology applied and the main results presented are reported in an effort to show the variety of the procedures followed. An important observation is that the multiple approaches implemented make a comparison between different works almost impossible, as the parameters reported cannot be compared in a straightforward way. Based on a careful study of the research published, a workflow of kinetic steps, applied on the initial experimental data, would be helpful and instructive, so as to obtain parameter values easily interpreted and compared. This perspective will be discussed in the last section of this article.

In Cánovas et al. 2020, ascorbic acid degradation was determined in three types of commercial fruit-based baby foods, having different low-moisture contents and stored at four different isothermal conditions—namely, at 5, 25, 30 and 40 °C [105]. The authors used a zero-order equation (Equation (3)) for ascorbic acid loss vs. storage time and a linear relationship between the ascorbic acid concentration and temperature.

Yeasmin et al. 2021 studied the Vitamin C retention during room/chill/frozen temperature storage in carambola, assuming a first-order degradation reaction (Equation (1)) [47]. As far as the secondary model applied, the Arrhenius equation was used to describe the temperature effect on the reaction rate (Equation (2)).

From the data provided in this research and a meta-analysis on the Vitamin C loss rates at a range of −18–20 °C, applying the Arrhenius equation for the calculation of *E_a_*, a value of *E_a_* close to 10 kJ/mol, was estimated; however, from the fitting adequacy results of lnk vs. 1/T (e.g., R^2^), it is obvious that a single Arrhenius equation throughout all this range may not be correct, since the underlying mechanism of Vitamin C degradation may not be the same. This behaviour, which frequently causes a “break” of the Arrhenius curve, has been also noticed and discussed in Giannakourou and Taoukis 2003 [96].

Temperature is not the only factor affecting Vitamin C degradation during storage. The effect of storage of fresh-cut strawberries under high-oxygen atmospheres (80 kPa O_2_) on the Vitamin C loss rates was kinetically studied at a temperature range (5–20 °C) and found to follow a first-order reaction (Equation (1)) [8]. In this work, a non-Arrhenius approach was implemented, and a secondary model proposed by Peleg et al. 2002 was used (Equation (13)) [106]:

(13) $$k=ln{\left(1+exp\left[c\cdot \left(T-T_{c}\right)\right]\right)}^{m}$$

where *T* is the storage temperature, *T_c_* is the limit value where the Vitamin C change showed accelerated rates and *c* (in K) and *m* (dimensionless) are constants of the Equation. Based on the secondary model used, the authors calculated the parameters of Equation (4)—namely, *c*, *T_c_* and *m*—to be equal to 1.0 × 10 − 2 per K, 284 K and 6.6, respectively, signalling the temperature of approximately 11 °C to be the critical temperature that abruptly promotes Vitamin C degradation. The relatively low value of this temperature implies that this nutritional factor (ascorbic acid) is probably more susceptible to small temperature increments than the other antioxidant compounds examined in this work (anthocyanins and antioxidant capacity).

In Van Bree et al. 2012, a practical tool was proposed through developed complex primary models to predict the ascorbic acid content in fruit juices during storage based on measurements of the residual headspace oxygen concentration after packaging, the oxygen permeability of the packaging material and the initial Vitamin C concentration [13]. The authors presented a detailed kinetic analysis of the influence of different initial headspace O_2_ concentrations on the oxidation of ascorbic acid and the subsequent formation and breakdown of DHAA, using irreversible and reversible models apart from the simplified zero- and first-order equations. As a result, a linear relationship was established between the degradation constants of ascorbic acid and the initial headspace oxygen concentrations.

Tsironi et al. 2017 presented a comprehensive shelf-life study for the storage of fresh cut salads under isothermal chill conditions (2.5–10 °C), where a Vitamin C loss followed a first-order decay and the Arrhenius law was used as the secondary model (*E_a_* = 122.6 ± 7.5 kJ/mol and *k_ref_* (*T_ref_* = 4 °C) =) 0.0178 ± 0.0013 (days^−1^) [107]. The kinetics developed were then successfully validated under non-isothermal conditions through an extended field test for a total duration of 118–147 h, depending on the different distribution scenarios applied. In Reference [7], a kinetic study of the cold storage of orange juice in polypropylene bottles, pre-treated either by conventional pasteurisation or by a high pressure step (HHP), was presented, and the relative shelf life was estimated (assuming a 50% loss as the acceptability limit). In that work, the Vitamin C loss was found to follow a first-order decay, and the Arrhenius law was used as a secondary model, with *E_a_* = 61.1 and 43.8 kJ/mol for the HHP and conventionally pasteurised samples, respectively.

Especially regarding frozen storage, there are few studies that have presented a systematic kinetic study of Vitamin C degradation in a wide range of frozen temperatures, and most of them were reported in the review article of Giannakourou and Taoukis 2019 [17]. In Giannakourou and Taoukis 2003, a shelf-life test was conducted at a −3 to −20 °C range, and the temperature dependence of Vitamin C loss was adequately modelled by the Arrhenius model, with the activation energy being calculated between 98 and 112 kJ/mol for frozen green peas, green beans, okra and spinach [15]. A pseudo-first-order reaction rate was used as the primary model (Equation (1)), whereas the Arrhenius, as well as the WLF models (similar to Equation (10), with reaction rates *k* instead of the *D*-values) were alternatively applied to describe the effect of temperature on the Vitamin C loss rate. Apart from validating the developed models under non-isothermal conditions simulating the real cold chain, a main conclusion of this research was that the different properties of the plant tissues significantly affect the rate of Vitamin C loss, with frozen spinach being the most susceptible to nutritional degradation and okra exhibiting a substantially lower loss rate. The same observation was stressed in an overview of the shelf-life prediction in the frozen fruits and vegetable chain, where the results on the kinetic parameters of different plant matrices and the corresponding estimations of their shelf lives at −18 °C revealed the crucial impact of the properties of the raw materials [17]. In this research paper, a stochastic kinetic approach was also proposed, aiming at incorporating the calculated uncertainty of model parameters when predicting the remaining shelf life of frozen food at any point within the real cold chain.

A similar kinetic study was implemented by Dermesonlouoglou et al. 2016 for frozen strawberries, where a preliminary step of osmotic treatment (using glucose, oligofructose and a high DE maltodextrin as the osmotic agents) was performed in an effort to improve the fruit stability during the subsequent frozen storage [10]. The activation energies estimated *E_a_* for the whole temperature range studied (from −5 to −16 °C) were between 120 and 185 kJ/mol, and the results on the rates of Vitamin C loss, especially at the lowest temperatures revealed, the protective role of the osmotic step applied. Similarly, in Giannakourou and Taoukis 2003, osmo-dehydrated frozen green peas were kinetically studied during frozen storage in terms of the Vitamin C loss, and the cryo-protective role of solute impregnation was confirmed [96]. The activation energies estimated using the Arrhenius secondary model, *E_a_*, for the different types of frozen tissues (control and osmo-dehydrated) were in the range between 75 and 95, whereas a slightly modified WLF equation was also adequately applied. In Table 2, some representative publications on the kinetic analysis of Vitamin C loss in frozen matrices is presented, also showing the equations used and some main results on the estimation of the kinetic parameters.

### 6.3. Effect of Processing and Storage on Vitamin C Loss

When addressing the overall nutritional degradation, during the processing and the post-processing handling, it is important to distinguish the individual impacts of each stage of the whole product lifecycle, from the stage of raw material to the final consumption. Despite what is generally expected, most of the studies that addressed this question concluded that the processing step may have a less significant effect on Vitamin C loss compared to the subsequent storage period impact, with the food matrix per se and the specific packaging and processing/storage conditions playing a crucial role.

**Figure 3:**
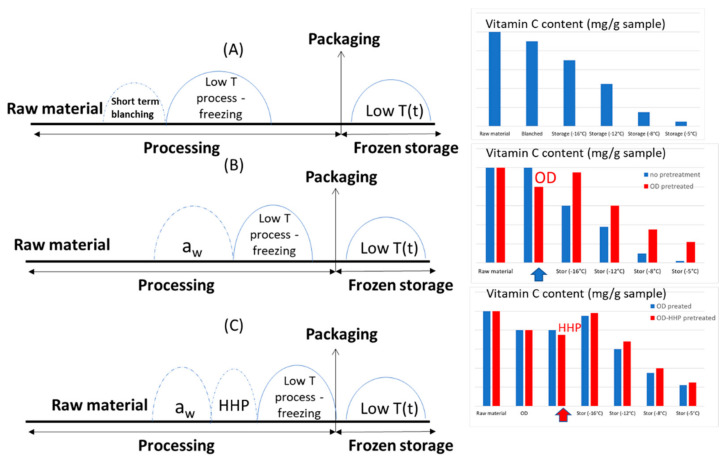
Indicative preservation scenarios (on the left, **A**–**C**) for frozen strawberries, depicted as successive hurdles (where a_w_ represents a water decrease processing step-namely, osmotic dehydration—and HHP is a high hydrostatic pressure process) and the corresponding Vitamin C content after each step (representative values based on the data published in References [10,114]).

From a hurdle technology perspective, it would be useful to design the integrated process and post-processing procedure as a sequence of preservation steps designed to assure product stability and safety. However, each “hurdle” may have a specific negative impact on sensitive compounds, such as Vitamin C, and the extent of this nutritional degradation usually depends on the specific conditions applied. As an example, in Figure 3, the impact of some pre-treatments on Vitamin C retention, prior to frozen storage, is depicted in an effort to design a multi-stage procedure for maximising the product nutritional quality at the time of consumption. To obtain more realistic results, the effects of the most important factors on the Vitamin C content for each processing/post-processing step should be quantified and mathematically described through the appropriate kinetic models. In the case study of Figure 3 (alternative treatments prior to the frozen storage of strawberries, data obtained from Dermesonlouoglou et al. 2016 [10] and Nunez-Mancilla et al. 2013 [114], the conditions of blanching (A), duration and temperature of the osmotic dehydration step (B), aiming at decreasing the product a_w_ and/or temperature/pressure/time of a high hydrostatic pressure treatment, could be optimised so as to achieve the maximum Vitamin C retention. In the example demonstrated in this figure, it is obvious that the application of a pre-treatment may cause a Vitamin C decrease (for example, during the osmotic dehydration step due both to leakage and oxidation, depending on the temperature applied), but this negative effect is usually more than counterbalanced by an improved stability during the subsequent frozen storage.

The Vitamin C degradation of strawberry puree was monitored after pasteurisation (at temperatures lower than 100 °C for less than 5 min) and subsequent storage at 35 °C for 14 d, with the results revealing small losses (below 10%) after the heat processing step, whereas a short storage of 4 days caused a significant loss of up to 76% [37]. In this work, Vitamin C was found to completely degrade after 14 d of storage, with the aerobic path being the most crucial mechanism leading to this decline. Similarly, in Reference [115], the impact of different intensities of heat treatment (performed at 70, 90 and 128 °C) and different types of storage (frozen-thawed, refrigerated and ambient storage) were investigated in the case of kale puree. All types of heat treatment were found to cause a substantial loss of Vitamin C (84–94%), which was further degraded during storage.

In Inanoglu et al. 2021, a high-pressure treatment and a microwave-assisted pasteurisation process were performed on green beans, followed by cold storage [116]. The results showed a significant loss (up to 40%) during processing, possibly due to the long process time (close to 10 min), with an equally or worse decline during cold storage, where almost all the Vitamin C was lost after 2 days of storage at 10 °C, mostly attributed to the presence of oxygen in the headspace of the pouches. The extension of the shelf life of reconstituted orange juice after high hydrostatic pressure (500 MPa, 35 °C, 5 min) pasteurisation over the conventional thermal (80 °C, 30 s) treatment was shown by Polydera et al. 2003, with HHP-treated juice having a longer durability of 11–65% when compared to the traditionally pasteurised counterpart [7]. Similarly, the advantageous effect of HHP treatment on the Vitamin C retention of orange juice was also mathematically demonstrated in Reference [6].

In a study on the stability of garlic mash potatoes, it was shown that the initial heat treatment, either in retort or a microwave-assisted thermal treatment, caused a Vitamin C loss of about 15%, while the subsequent storage under abusive conditions (at 38 °C, accelerated shelf-life testing) caused a more severe degradation of about 35% after 6 months of storage [42]. In Chen et al. 2020, a high hydrostatic pressure treatment (400–600 MPa for 5–15 min) was performed as an alternative to a heat treatment (85 °C for 10 min) on a kiwi pulp beverage, and the results showed that both methods caused a significant Vitamin C loss, ranging from a 45 to 75% decline [117]. The authors showed that the pressure–time conditions in HHP processing played a crucial role, with the combination of 400 MPa at 15 min providing the maximum retention immediately after processing and during the subsequent chill storage. Sroy et al. 2019 studied the impact of ozone treatment as an alternative to the traditional pasteurisation process on the Vitamin C content of Cantaloupe melon juice and, also, during refrigerated storage [81]. Vitamin C was much better retained in ozone-treated juices when compared with the pasteurised ones (68 vs. 39%, respectively), a positive effect also being observed during cold storage.

## 7. Alternative Kinetic Approaches for Describing the Effect of Process and/or Storage on Vitamin C Retention

Based on the previous findings on vitamin loss rates and the effects of the prevailing food/process/storage parameters, it can be concluded that the development of an integrated kinetic model is crucial for a better understanding the kinetics of this degradation. In the literature, there are several approaches proposed for the construction of such models, with the two-step, deterministic procedure being the most popular one in the earlier publications and a more complicated methodology based on Bayesian and probabilistic statistics (using the Monte Carlo scheme) being frequently applied in the recent literature. Alternative kinetic methodologies are explicitly described in References [16,118], with some examples being presented in detail. A profound analysis, as well as the pros and cons of each approach, was explicitly presented by Van Boekel 1996, 2008, 2021 and 2020 [119,120,121,122]. Since the goal of modelling is to predict the future behaviour of a specific food matrix as accurately and precisely as possible, it is necessary to incorporate and effectively fit experimental data to mathematical models, and in this task, appropriate statistics is needed to estimate and take into account the uncertainties involved.

**Figure 4:**
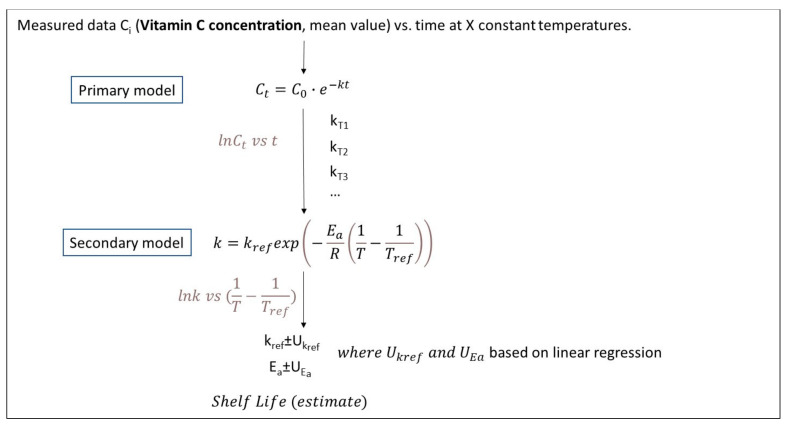
Indicative workflow of the conventional two-step approach (frequentist approach) using an example of the Vitamin C loss during frozen storage for vegetables, providing a mean value for the prediction of the shelf life.

**Figure 5:**
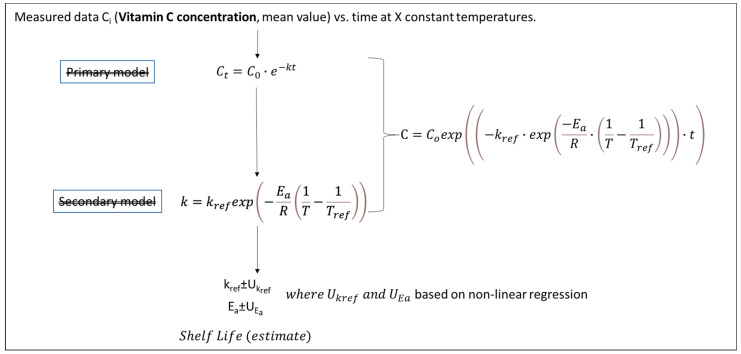
Indicative workflow of the global (one-step) approach (frequentist approach) using an example of the Vitamin C loss during frozen storage for vegetables, providing a mean value for the prediction of the shelf life.

**Figure 6:**
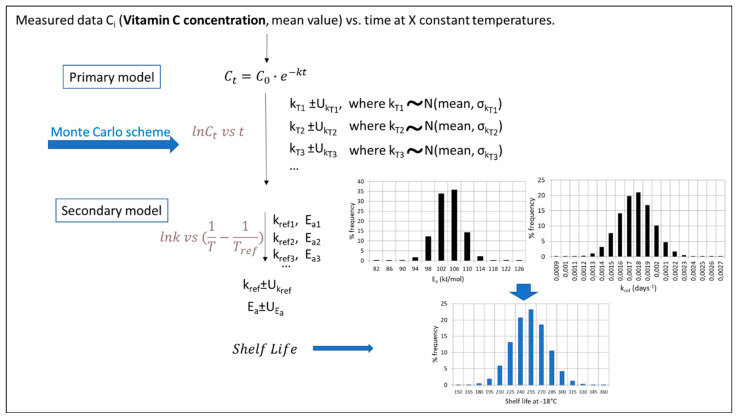
Indicative workflow of a stochastic approach (based on a two-step analysis) using an example of the Vitamin C loss during frozen storage for vegetables, providing a distribution curve for the prediction of the shelf life.

**Figure 7:**
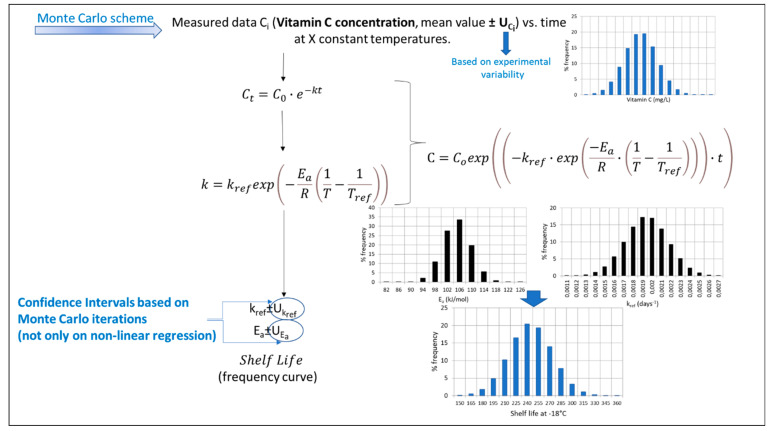
Indicative workflow of a stochastic approach (based on a one-step analysis) using an example of the Vitamin C loss during frozen storage for vegetables, providing a distribution curve for the prediction of the shelf life and taking into account the variability of the experimental data (Vitamin C concentration at different storage times and at different storage temperatures in the frozen range).

The main steps of the algorithm used in each procedure (based on experiments under a constant temperature), along with the equations used in the case of Vitamin C degradation, are schematically depicted in Figure 4, Figure 5, Figure 6 and Figure 7. Briefly, in Figure 4, the traditional two-step analysis is shown, applied in the majority of early works on frozen vegetable quality loss during storage (as shown in Table 2). According to this scheme, in the first step, the Vitamin C concentration is measured at different storage times, and the rate of the loss (k_VitC_) is estimated at selected constant temperatures through an appropriate primary model (usually a first-order decay). In a second step, the temperature dependence of the degradation rate is described through the use of a particular secondary model, with the Arrhenius equation being the most popular one. In an alternative approach (Figure 5), the kinetic parameters (in our case, *E_a_* and *k_ref_*) can be determined in a single step from the same dataset at isothermal conditions (nonlinear regression analysis), considering the available raw data as a whole by incorporating the secondary model equation into the mathematical expression of the primary model. The outcome of those two deterministic approaches is, roughly speaking, the estimates (mean values) of the kinetic parameters, followed by an error, which are based on specific assumptions [121,122] and are derived from different calculations in each case. The result is that the uncertainty estimated when applying the two-step approach, calculated via a regression analysis, is usually wider than the one calculated with a global one-step approach [16,123]. Although, in both cases described so far, it is possible to estimate the joint confidence intervals [124,125], the predictions provided by those deterministic models would statistically represent the mean of the repeated measurements [126].

In an alternative to frequentist methodology, the basic principle behind the calculations is that kinetic parameters cannot be efficiently described by a single value, as this approach does not exploit the whole information obtained from either the mathematical models or from the raw data initially obtained through isothermal experiments. Therefore, this stochastic approach involves the application of a Monte Carlo simulation scheme, assuming that the *E_a_* and *k_ref_* variabilities are described by a certain distribution (in Giannakourou and Taoukis 2020, a Gaussian distribution is used for that purpose [16]). The outcome of this analysis, when the predictions are performed, is a frequency curve instead of a single value ±95% confidence interval, providing more realistic results (Figure 6). In a final fine-tuning of this latter stochastic approach, the information of the variability of the raw (experimental) data can also be incorporated within the analysis, according to Bayesian statistics, that apply, for this purpose, the Monte Carlo iterative procedure (Figure 7). By doing so, the resulting calculations on the predicted factor (in our case, the shelf life of a product at a certain temperature) are described by a distribution rather than a value, which is expected to be different than the previous one (when accounting only for the uncertainty of kinetic parameters and not for the variability of the initial concentration measurements), mimicking, in a more reliable way, the real uncertainty of quality retention and, thus, the shelf life. Van Boekel 2021 in Reference [121] presented a similar workflow based on a Bayesian approach to obtain the probability distributions of parameters rather than point estimates by using a published dataset on L-carnitine inactivation under isothermal processing conditions [127].

As an example of the implementation of these different algorithms for the prediction of a frozen vegetable shelf life (green peas), based on a 50% Vitamin C loss, the classical two-step analysis derived the following results for the kinetic parameters applied—namely, *E_a_* = 102.31 ± 17.91 kJ/mol and *k_ref_* = 0.00196 ± 0.000795 d^−1^—leading to a shelf life of 248 d at −18 °C (mean value) [16]. When a global, one-step approach was assumed, taking into account all the data in one nonlinear analysis (MATLAB), the corresponding values for the parameters of a single, integrated equation (Figure 5) were *E_a_* = 104.24 ± 11.34 kJ/mol and *k_ref_* = 0.00177 ± 0.000494 d^−1^, and the frozen green peas shelf life was estimated at −18 °C for approximately 250 d (mean value). Based on the latter scheme and the 95% confidence intervals of *E_a_* and *k_ref_* calculated (described by the corresponding distribution curves depicted within Figure 6), a stochastic algorithm was implemented based on a Monte Carlo analysis and a code developed in the FORTRAN algorithm (Figure 6). The results for the shelf-life (SL) prediction at −18 °C, including its uncertainty, were described by a frequency curve rather than a single estimate, with a mean value (SL estimate) ±95% CI equal to 252.6 ± 40.4 days, giving a more realistic prediction than the single value estimation of 250 d. Going a step further, the significant variability of the raw data (Vitamin C concentration measurements) was introduced into the final predictions using the stochastic approach depicted in Figure 6, where a more sophisticated Monte Carlo algorithm was implemented and a one-step analysis was carried out. The corresponding results for the shelf-life (SL) prediction at −18°C were described by a normal distribution curve with a similar mean value but a larger ±95% CI equal to 249.3 ± 56.8 days, respectively. At this point, it should be stressed that the workflows presented (Figure 4, Figure 5, Figure 6 and Figure 7) have a generalised application and are not limited either to the Vitamin C quality factor or to the kinetic models used in this example. They may be slightly modified so as to include different primary/secondary models and data on different quality indices or even be implemented based on data under non-isothermal conditions.

## 8. Discussion and Concluding Remarks

Being an essential nutritional element, the decrease of the Vitamin C concentration to levels unacceptable by the legislation or industrial practice often renders this compound a crucial indicator for product quality and, thus, is used for determining the shelf life of foods such as juices, frozen foods and vegetables and dehydrated products of plant origin. In food science and engineering literature, the modes and rates of Vitamin C loss have been extensively studied, and nutritional degradation has been shown to significantly depend not only on the processing/storage conditions and packaging but, also, on the food properties.

The effects of conventional and novel preservation processes on Vitamin C were systematically approached and assessed in order to allow for a quantitative benchmarking of the different processes. These included the mainstream preservation methods based on thermal processing, low temperature and moisture reduction, as well as high-pressure processing, ultrasound, pulsed electric fields, cold plasma and pulsed light.

As an overall conclusion of this critical review, novel, less intensive techniques, or a combination of successive treatment steps, are emerging and being proposed in order to mitigate the negative effects of the traditional preservation processes. Another important observation underlined in many studies is that, assuming that the processing is appropriately designed so as to cause the least nutritional degradation (either by combining milder treatments or by optimising a particular preservation technique), the post-processing period, including handling, distribution and storage, may be the most determinant factor for the Vitamin C overall decline. Regarding the impact of this stage of the whole lifecycle of the product, there is a significant body of literature that also includes systematic kinetic studies that highlight the important impact of the actual conditions of the chill and frozen distribution chains, factors difficult or even impossible to completely control [16]. In many cases, the uncertainty of the shelf-life predictions, which is the main outcome of any kinetic analysis implemented, and the uncontrollability of the factors that are part of the kinetic model structure are approached by more sophisticated mathematical and statistical tools. The gradual evolution of the kinetic analysis, from the basic two-step frequentist methodology to the stochastic approach, may provide more realistic predictions, allowing for the fine-tuning of the processes and handling procedures.

## Figures and Tables

**Table 2:** Overview of the kinetic methodology applied and the main results obtained for the storage effect for various frozen fruits and vegetables.

Frozen Matrix Studied and Temperature Range Studied	Indicative Value of Vitamin C Initial Content (I.C.) and Extent of Loss at Different Temperatures	Primary and Secondary Models Used	*E_a_* (kJ/mol) *T_ref_* (°C), *k_ref_* (d^−1^)	Reference
Spinach (−5 to −18 °C)	I.C. = 25.9 mg/100 g of frozen product % loss (after 100 d of storage): ≈70% (−12 °C) <20% (−18 °C)	1st order Equation (1) Arrhenius model: Equation (2)	132.0 ± 5.80 −18 °C (0.0029 ± 0.0004)	[108]
Spinach (−5 to −18 °C)	I.C. = 31.1 mg/100 g of frozen product % loss (after 75 d of storage): ≈92% (−12 °C) ≈20% (−20 °C)	1st order: Equation (1) Arrhenius model: Equation (2)	120.0 ± 13.73 −20 °C (0.00365 ± 0.0012)	[15]
Green Beans (−6 to −18 °C)	I.C. = 146.1 mg/100 g of frozen product % loss (after 4 d of storage): ≈20% (−6 °C)	1st order: Equation (1) Arrhenius model: Equation (2)	42.0 −15 °C 0.03226	[109]
Green Beans (−1 to −16 °C)	I.C. = 25.3 mg/100 g of frozen product % loss (after 100 d of storage): <50% (−12 °C) ≈30% (−16 °C)	1st order: Equation (1) Arrhenius model: Equation (2)	101.5 −20 °C 0.00223	[15]
Broccoli (−7 to −25 °C)	I.C. = 32.9 mg/100 g of frozen product % loss (after 100 d of storage): <85% (−15 °C) ≈72% (−25 °C)	1st order: Equation (1) Arrhenius model: Equation (2)	60.24 ± 7.20 −15 °C (6.80 ± 0.69) ∗ 10^−3^	[110]
Green Peas (−1 °C to −16 °C)	I.C. = 28.5 mg/100 g of frozen product % loss (after 100 d of storage): ≈40% (−12 °C) <25% (−16 °C)	1st order: Equation (1) Arrhenius model: Equation (2)	97.9 ± 9.60 −20 °C 0.00213	[15]
Okra (−1 °C, to −16 °C)	I.C. = 25.3 mg/100 g of frozen product % loss (after 100 d of storage): ≈50% (−8 °C) <20% (−16 °C)	1st order: Equation (1) Arrhenius model: Equation (2)	105.9 −20 °C 0.00106	[15]
Watercress (−7, −15 and −30 °C)	I.C. = 37.6 mg/100 g of frozen product % loss (after 100 d of storage): ≈83% (−7 °C) <75% (−30 °C)	1st order: Equation (1) Arrhenius model: Equation (2)	24.73 ± 4.52 −15 °C (4.32 ± 0.45) * 10^−3^	[111]
Pumpkin (−7, −15 and −25 °C)	I.C. = 11.0 mg/100 g of frozen product % loss (after 100 d of storage): ≈64% (−15 °C) <45% (−25 °C)	1st order (fractional model): $C=C_{eq}+\left(C_{0}-C_{eq}\right)e^{-k_{vitC}t}$ Arrhenius model: Equation (2)	41.39 ± 7.22 −15 °C (25.50 ± 6.40) ∗ 10^−3^	[112]
Strawberry (−5, to −16 °C)	I.C. = 29.3 mg/100 g of frozen product % loss (after 100 d of storage): ≈60% (−12 °C) <30% (−16 °C)	1st order: Equation (1) Arrhenius model: Equation (2)	123.90 ± 8.10 −18 °C 0.003 ± 0.0004	[10]
Kiwi (−5 to −25 °C)	I.C. = 24.6 mg/100 g of frozen product % loss (after 100 d of storage): ≈25% (−15 °C)	1st order: Equation (1) Arrhenius model: Equation (2)	81.16 ± 9.48 −18 °C 0.0055 ± 0.0010	[9]
Carrot (−7, −15 and −25 °C)	I.C. = 11.1 mg/100 g of frozen product % loss (after 120 d of storage): ≈50% (−18 °C)	1st order: Equation (1) Arrhenius model: Equation (2)	21.24 ± 1.58 −15 °C (6.66 ± 0.25) ∗ 10^−3^	[113]
